# 337. Diagnostic Yield and Impact on Antimicrobial Management of 16s rRNA Testing on Clinical Specimens

**DOI:** 10.1093/ofid/ofac492.415

**Published:** 2022-12-15

**Authors:** Pruke Eamsakulrat, Angsana Phuphuakrat, Pitak Santanirand

**Affiliations:** Ramathibodi Hospital, Mahidol University, Bangkok, Krung Thep, Thailand; Ramathibodi Hospital, Mahidol University, Bangkok, Krung Thep, Thailand; Ramathibodi Hospital, Mahidol University, Bangkok, Krung Thep, Thailand

## Abstract

**Background:**

16s rRNA gene sequencing has an advantage over traditional bacterial culture in situations where bacteria are difficult to culture, unculturable, or have previously been exposed to antimicrobial. The study on its applicability to direct clinical specimens is still limited. We studied the value of 16s rRNA gene sequencing from direct clinical specimens on antimicrobial management.

**Methods:**

Inpatient adults whose attending physician requested 16s rRNA gene sequencing and corresponding bacterial culture from a direct clinical specimen were prospectively included between January and December 2021. Diagnostic yield and the impact of 16s rRNA gene sequencing on antimicrobial management were investigated.

**Results:**

A total of 434 specimens from 374 patients were requested. All patients had a complete follow-up and had a definitive final diagnosis. The sensitivity and specificity of 16s rRNA gene sequencing were 38.3% and 93.9%, respectively. The agreement between 16s rRNA gene sequencing and conventional culture among specimens obtained from bacterial infection cases was 83.8% (the κ coefficient 0.664, p< 0.001). Among bacterial infection cases, the proportion of 16s rRNA gene sequencing positive/culture-positive was 32.4% (82 specimens), and the proportion of sequencing positive/culture-negative was 5.9% (15 specimens). The impact on antimicrobial management occurred in 10 (2.3%) specimens, which all resulted in the continuation of antibiotics. An impact on antimicrobial management was highest in the skin and soft tissue infection, followed by bone and joint infection. In contrast, CAP/HAP/VAP was less likely to benefit from this test. Long turnaround time was the limitation of the test in this study, the median (interquartile range) was 11 (9-13) days in the 16s rRNA gene sequencing positive group, and 2 (1-4) days in the 16s rRNA gene sequencing negative group.

**Conclusion:**

The overall diagnostic yield of 16s rRNA gene sequencing in bacterial infection cases was fair. 16s rRNA gene sequencing was useful in selected cases, but it cannot replace traditional culture. The negative result did not exclude bacterial infection. Restrictions on test requests may improve test utilization in this setting.

**Disclosures:**

**All Authors**: No reported disclosures.

